# Convalescent Plasma Transfusion for the Treatment of COVID-19 in Adults: A Global Perspective

**DOI:** 10.3390/v13050849

**Published:** 2021-05-07

**Authors:** Saly Kanj, Basem Al-Omari

**Affiliations:** College of Medicine and Health Sciences, Khalifa University of Science and Technology, Abu Dhabi 127788, United Arab Emirates; saly.elkanj@ku.ac.ae

**Keywords:** COVID-19, convalescent plasma transfusion, therapeutics, clinical trials

## Abstract

More than one year into the novel coronavirus disease 2019 (COVID-19) pandemic, healthcare systems across the world continue to be overwhelmed with soaring daily cases. The treatment spectrum primarily includes ventilation support augmented with repurposed drugs and/or convalescent plasma transfusion (CPT) from recovered COVID-19 patients. Despite vaccine variants being recently developed and administered in several countries, challenges in global supply chain logistics limit their timely availability to the wider world population, particularly in developing countries. Given the measured success of conventional CPT in treating several infections over the past decade, recent studies have reported its effectiveness in decreasing the duration and severity of COVID-19 symptoms. In this review, we conduct a literature search of published studies investigating the use of CPT to treat COVID-19 patients from January 2020 to January 2021. The literature search identified 181 records of which 39 were included in this review. A random-effects model was used to aggregate data across studies, and mortality rates of 17 vs. 32% were estimated for the CPT and control patient groups, respectively, with an odds ratio (OR) of 0.49. The findings indicate that CPT shows potential in reducing the severity and duration of COVID-19 symptoms. However, early intervention (preferably within 3 days), recruitment of donors, and plasma potency introduce major challenges for its scaled-up implementation. Given the low number of existing randomized clinical trials (RCTs, four with a total of 319 patients), unanticipated risks to CPT recipients are highlighted and discussed. Nevertheless, CPT remains a promising COVID-19 therapeutic option that merits internationally coordinated RCTs to achieve a scientific risk–benefit consensus.

## 1. Introduction

Multiple cases of acute respiratory syndrome with unclear precursors were recorded during December 2019 in Wuhan, the capital of Hubei province in central China. Subsequent analysis of samples from patients’ lower respiratory tract indicated the presence of an unprecedented strain of human coronavirus, referred to as the severe acute respiratory syndrome coronavirus-2 (SARS-CoV-2) [1,2,3], and its corresponding disease was dubbed coronavirus disease 2019 (COVID-19). Since the World Health Organization (WHO) declared the COVID-19 outbreak as a global pandemic on 11 March 2020, the cumulative number of global confirmed cases has surpassed 142 million with over 3 million deaths as of 21 April 2021 [4]. In response to growing pressures from the pandemic, more than 2000 clinical trials have been actively implemented during 2020, yet a unified treatment approach has not been recognized by the global healthcare and scientific communities to date [5]. Adding to the complexity of the current situation, government bodies and regulatory agencies overseeing national healthcare response plans have been subject to an unprecedented rate of trial reports published by multiple sources with often conflicting findings.

Global- and national-scale recovery timelines remain unclear amidst the uncertainty in treatment approaches and the emergence of new COVID-19 mutant strains [6]. Standard supportive care guidelines released by the WHO remain the status quo and mainly include symptom management through assisted ventilation and fluid management [7]. Remdesevir has been the only repurposed drug authorized by the United States (US) Food and Drug Administration (FDA) solely for emergency use [8]. Other drugs such as Hydroxychloroquinine [9], Ivermectin [10], and Oseltamivir [11] have been reportedly found to be comparably effective to standard supportive care for COVID-19 patients. Nevertheless, the generalized effectiveness of repurposed drugs remains subject to debate in the absence of large-scale data. As such, international efforts have been focused on vaccine development, which is naturally the product of several years of research and clinical trials before being authorized for general use.

The multi-phase vaccine development process encompasses pre-clinical testing (non-human trials), safety and dosage assessments (Phase 1), expanded safety trials (Phase 2), scalable efficacy trials (Phase 3), limited use authorization, and ultimately, full-scale approval. Given the urgency of the COVID-19 pandemic, a total of 89 vaccines are currently in human clinical trials—many of which are combining Phase 1 and 2 trials—while 27 have reached the final stages of testing [12]. Despite the timely progress in vaccine developments, two dynamic challenges hinder their post-efficacy stage for the global population. The primary set of challenges, especially for developing countries, lies in the supply chain and logistics in terms of scaled-up manufacturing and quality control, regional and local coordination of supply, and equality in distribution and governmental subsidies [13,14]. These factors exclusively stem from the post-efficacy stage, presumably after addressing any lurking uncertainties, such as the optimal timing and dosage of booster vaccine shots. Another transient challenge lies in the ongoing emergence of new SARS-CoV-2 variants [6,15]. The coronavirus genome is highly susceptible to mutations [16], which increases the likelihood of variant forms to evolve with time—a process termed genetic drift [17]. Hence, there is a sizable risk that a newly mutated virus might not be recognized by vaccine-acquired immune recognition from pre-mutation genomes.

Based on the above uncertainties in the COVID-19 response, numerous healthcare practitioners across the world have resorted to the conventional approach of convalescent plasma transfusion (CPT) therapy [18]. CPT therapy was first proposed in the late 1800s to treat a variety of previously unknown infectious diseases that lacked conclusive treatment options [19,20]. The general concept of CPT is based on the notion of relaying passive immunity by screening blood samples taken from a recovered individual (donor) for specific neutralizing antibodies, which are then administered to a patient suffering from the same disease to ameliorate symptoms and reduce mortality [21]. CPT has been particularly effective when used for diseases targeting the respiratory system, including the severe acute respiratory syndrome (SARS), Middle East respiratory syndrome (MERS), and influenza (H5N1 and H1N1) outbreaks [22].

Investigating the efficacy of CPT for treating COVID-19 patients has been recommended by the FDA since March 2020 [23]. Consequently, local-scale randomized clinical trials (RCTs), matched-control studies (MCSs), and case reports (CRs) conducted during 2020 offered preliminary evidence on the safety and efficacy of CPT therapy for COVID-19 patients [24]. However, the findings are subject to debate within the healthcare and scientific communities due to the lack of concrete evidence from statistically representative large-scale RCTs [25]. For instance, some studies were terminated early while reporting inconclusive results and a non-significant correlation between CPT and clinical improvement [26].

COVID-19 infections are generally mild and/or asymptomatic in the majority of the pediatric population with less than 2% of COVID-19 cases reported for infant and child age groups [27,28,29,30]. Additionally, current recommendations for the treatment of severe COVID-19 in children is limited to monitoring and supportive care [31]. Consequently, very few studies on convalescent plasma transfusion (CPT) therapy for COVID-19 pediatric patient groups are available to date. Rodriguez et al. [32] and Shankar et al. [33] provide case reports on individual high-risk patients with pre-existing conditions, namely, congenital heart disease (6 weeks old; female) and lymphoblastic leukemia (4 years old; female), respectively. In both cases, patients show a considerable reduction in symptoms after CPT therapy, which suggests a positive clinical correlation in infants and children with underlying conditions. Moreover, Figlerowicz et al. [34] reports the first attempt of CPT therapy on a 6-year-old child diagnosed with COVID-19-induced aplastic anemia without pre-existing conditions. Following the administration of antiviral drugs without signs of recovery during the first 5 weeks, a single dose of convalescent plasma (200 mL; 1:700 titer) resulted in recovery from COVID-19 within 3 weeks, while hematologic deficiencies persisted. The most representative attempt involving COVID-19 pediatric patients to date is the recent work of Małecki et al. [35]. Their study included a group of 13 patients (median age of 12 years), of which six CPT-recipient patients recovered within 3 days of transfusion, while the remaining seven control patients recovered after 12 days. Based on the limited data and a gap period of 10 days between infection and transfusion, the study suggests CPT may be a promising treatment for COVID-19 in children. Given the limited data on pediatric patients, underlying conditions, and/or notable lag times in administering CPT, the aforementioned studies were excluded from the review to avoid introducing significant biases within the pediatric age group.

The most recent reviews have been published before January 2021, including rapid and living systematic reviews and meta-analyses [25,36,37,38,39,40,41], after which several papers documenting trials on CPT as a treatment for COVID-19 have been published. Furthermore, previous reviews were largely based on limited RCT data. Taking into consideration the urgency of the topic and the struggle to identify treatments, it is vital that the literature is updated more often than in normal circumstances. Therefore, in this review, we conduct a global literature search of all studies examining the clinical effectiveness of CPT therapy for the treatment of COVID-19 and systematically analyze available data to date through a random-effects model. We also present a critical discussion on the limitations and challenges facing CPT therapy in the context of the ongoing COVID-19 response.

## 2. Materials and Methods

A comprehensive literature search was conducted by the first author (S.K.). The Cochrane Library, PubMed (MEDLINE), EMBASE, ScienceDirect, and Google Scholar were electronically searched from January 2020 until January 2021. The keywords related to COVID-19, CPT, and patients’ treatment concepts (“Convalescent”, “Plasma”, “Transfusion”, “COVID19”, “COVID-19”, “Coronavirus”, “SARS-CoV-2”, “Patient”, “Treatment”, “Passive”, and “Immunity”) were used with Boolean combinations.

No restriction on publication language was applied. In addition, the reference lists of articles resulting from the keyword search were manually inspected to identify additional relevant studies.

### 2.1. Eligibility Criteria

Studies were included in this review if they (1) are RCTs, MCSs, or CRs, which quantitatively report on CPT treatment for COVID-19 adult patients, (2) report trial data and performance metrics—in terms of the following parameters (at the minimum): number of patients, dosages, sampling periods, and mortality, and (3) report titers of SARS-CoV-2-neutralizing antibodies at a threshold therapeutic level of 1:80 [42,43]. Publications were excluded if they were conference proceedings, published as an abstract without the full text, or including patients under the age of 18 years old.

### 2.2. Data Synthesis

The clinical data were extracted and compiled from all studies and ingested into MATLAB^®^ R2020a [44]. Boxplots for each study category (RCT, MCS, and CR) were produced using the Descriptive Statistics and Visualization toolbox in MATLAB [45]. Commonly used for descriptive statistics, boxplots graphically depict datasets through their quartile ranges, while outliers are represented as individual points. Importantly for our analysis, boxplots are non-parametric and avoid prescribing a statistical distribution to an unknown sample population. Hence, it is a standardized tool to inter-compare datasets based on their minimum, maximum, median, and interquartile ranges, with the spacing of the latter indicating the degree of variance in the data. Nevertheless, boxplots provide a coarse representation of the aggregated datasets and fail to account for inherent biases and uncertainties across studies, which may alter the findings. As such, a random-effects model was implemented in MATLAB^®^ using the “LinearMixedModel” (LMM) embedded function [46], which returns estimates of the best linear unbiased predictors of random effects according to a prescribed significance level (5% used here). To ensure representative and generalized model output, a model ensemble was set up using three different methods for approximating the degrees of freedom (DOF), namely, the default constant definition, the Satterthwaite approximation, and the infinity definition [46,47].

## 3. Results

### 3.1. Included Studies

The literature search identified a total of 181 articles, of which 63 articles remained after removing duplicates. Following the title and abstract screening against the inclusion/exclusion criteria, 23 articles were excluded. During the full-text screening, one RCT article was excluded, since the majority of the trial’s CPT patients received plasma transfusions with non-detectable levels of SARS-CoV-2-neutralizing antibodies based on post-trial measurements (antibody titers < 1:80) [48]. The remaining 39 articles included in this review were geographically distributed across the following countries: China (13), USA (11), Mexico (2), South Korea (2), Turkey (2), Argentina (1), France (1), Hungary (1), Italy (1), Iran (1), Iraq (1), Netherlands (1), Qatar (1), and Spain (1).

### 3.2. Characteristics of Included Studies

The 39 studies included four RCTs [26,49,50,51], 14 MCSs [42,52,53,54,55,56,57,58,59,60,61,62,63,64], and 21 CRs [43,65,66,67,68,69,70,71,72,73,74,75,76,77,78,79,80,81,82,83,84]. The majority of studies (28) were published in peer-reviewed journals, while 11 studies were available as pre-prints. Despite still being under peer-review, the raw data reported in these pre-prints remain highly relevant to our analysis, regardless of the methodology and recommendations provided.

An important uniformity across the studies was that all patients were included on the basis of demonstrating severe or life-threatening complications associated with COVID-19. Hence, all patients received one or more form of concomitant supportive care including mechanical ventilation and/or repurposed drugs as per WHO and local healthcare guidance. Almost half the studies (20) applied an antibody inclusion threshold to donor samples before transfusion, while the remaining (19) did not apply any screening to samples. The volume of transfused plasma ranged from 200 to 1200 mL, while the post-CPT therapy follow-up occurred between 7 and 60 days. Patient cohort age groups ranged from 23 to 96 years with a higher male proportion in each study. The total number of patients per study ranged from 49 to 103 for RCTs, from 20 to 3166 for MCSs, and from 1 to 31 for CRs. Table 1 lists the main characteristics relevant to our analysis and the results presented in the following section.

### 3.3. Statistical and Random-Effects Model Results

**Figure 1:**
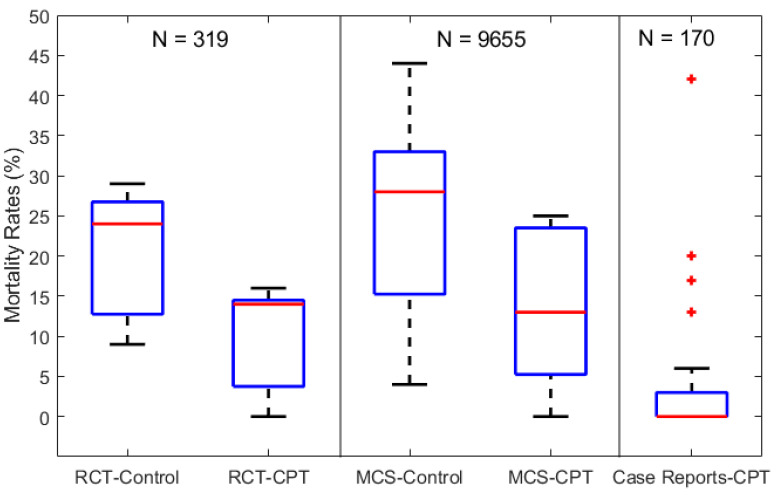
Boxplots of mortality rates in COVID-19 patients from control and CPT recipient groups of RCTs, MCSs, and CRs. The total number of patients included in each category of studies is labeled above as N = 319, 9655, and 170 for the RCTs, MCSs, and CRs, respectively.

Figure 1 shows the boxplots of mortality rates as reported from the 39 studies and aggregated per RCTs, MCSs, and CRs with a total patient count of N = 319, 9655, and 170, respectively. The mortality rates for both the control and CPT recipient groups are shown, except for the CR studies which, by definition, are not assigned a control group. The median mortality rates of the control groups (RCT-Control and MCS-Control) are shown to be comparable at 24 and 28%, respectively. However, the MCS-Control group records a higher variance with mortality rates reaching around 45% compared to the 30% limit of the RCT-Control groups. Similarly, the median mortality rates of the corresponding CPT groups (RCT-CPT and MCS-CPT) are comparable at around 14%, while the MCS-CPT group shows a higher variance with mortality rates reaching 25%, compared to the 16% limit reported by the RCT-CPT group. The 28-fold difference in total patient numbers between the RCT (319) and MCS groups (9655) explains the higher variance in mortality rates from the latter group.

The results from both the RCT and MCS groups in Figure 1 indicate an overall reduction of 46% in mortality rates (~26 to 14%) in patients receiving CPT therapy compared to the control groups receiving standard supportive care and/or repurposed drugs. On the other hand, the CR-CPT group shows significantly lower mortality rates (less than 5%) compared to the RCT and MCS groups, but with outliers reaching 43%. This reflects the potential of high bias when aggregating individual case reports. For instance, several CR studies consisted of COVID-19 patients with impaired immunities, which suppressed their response to standard supportive care [68,79,83]. However, their symptoms were significantly reduced and showed signs of recovery after 3–5 days of CPT with antibody titer levels higher than 1:80, compared to undetectable levels pre-CPT.

To address the risk of bias and generalize the boxplot results, a random-effects model was set up based on the parameters listed in Table 1. Mortality rates were derived based on the longest follow-up period in each study, and odds ratios (ORs) were aggregated for each of the RCT and MCS study categories (CRs do not have control groups). A meta-regression analysis based on the MATLAB LMM function [46] was used to evaluate the significance of the parameters listed in Table 1 (i.e., cohort age, follow-up duration, plasma volume/dose) with respect to mortality across the studies. Hypothesis testing using the two-tailed test at a 95% confidence interval (5% significance) was carried out.

The random-effects analysis showed median mortality rates of 11 vs. 23% for the CPT and control groups, respectively, across the four RCT studies with an OR of 0.41. This suggests that CPT was correlated with a 59% reduction in the likelihood of mortality among COVID-19 patients. This association was further observed based on the MCS random-effects model results, for which mortality rates of 21 vs. 32% for the CPT and control groups with an OR of 0.47 (i.e., 53% reduction in the likelihood of mortality). To further investigate these findings, an overall random-effects model was used to aggregate results from both RCT and MC studies. Similarly, mortality rates of 17 vs. 32% for the CPT and control groups were recorded, respectively, with an OR of 0.49—a 51% reduction in the overall likelihood of mortality across all trials. The meta-regression analyses indicated that cohort age and the follow-up duration did not affect the aggregate OR computed for all clinical trials.

## 4. Discussion

The FDA clearly states that the authorization of CPT for emergency use is not meant to replace ongoing clinical trials, since there is insufficient evidence to currently designate CPT as a new standard of care [23]. In this review, a total of 39 trials and case reports were analyzed and systematically inter-compared to assess the efficacy of CPT for the treatment of COVID-19 patients. Overall, the findings of this review support the efficacy of CPT therapy for the treatment of COVID-19 patients, particularly those experiencing severe symptoms. This outcome generally contests the findings of former systematic reviews on the efficacy of CPT therapy for COVID-19, with some reporting inconclusive results [26,37]. Nevertheless, several underlying risks and limitations may undermine the efficacy of CPT therapy in the context of COVID-19.

### 4.1. Plasma Potency: SARS-CoV-2 Antibody Inclusion Thresholds

As the primary active agents in convalescent plasma, neutralizing antibodies are considered the key indicator of plasma potency for CPT therapy. However, only two of the 39 studies explicitly reported a significant correlation between mortality and SARS-CoV-2-neutralizing antibody titer levels in CPT doses. Interestingly, despite the importance of verifying plasma potency, almost half the studies (18) did not screen donor plasma samples to ensure adequate levels of SARS-CoV-2 antibodies [49,53,54,55,60,62,63,67,69,70,72,74,75,76,80,81,82,84]. In one RCT trial in India by Agarwal et al. [48], the majority of patients (71%) received CPT doses with relatively low SARS-CoV-2-neutralizing antibody titer (<1:80), while the remaining patients received non-detectable levels. There is a consensus among trials to date that an adequate titer of neutralizing antibodies should exceed 1:80 [42,43]. Hence, the Agarwal et al. [48] RCT was excluded from the current review (and Figure 1), as it lacked the fundamental biological and therapeutic mechanism of CPT.

It is important to note that different serologic tests were used to report antibody levels across the 20 studies, which performed plasma screening (see Table 1). The different assay results included the enzyme-linked immunosorbent assay (ELSA) and neutralizing antibody (NA) indices (i) or titers (t), signal-to-cutoff ratio (S/Co), and total antibodies (TA) in arbitrary units per volume (AU/mL). Several types of assays have been developed in a short period of time in response to the COVID-19 outbreak, each with its own set of uncertainties in terms of clinical sensitivity and specificity, such as sample collection and handling, technological challenges, unknown systematic error margins, and dependencies on patient health conditions [85]. According to the most recent literature, a single assay result will not reflect a representative quantitative measure, whereas merging different test results can help minimize biases and provide more conclusive therapeutic thresholds [86,87].

### 4.2. CPT Intervention Time

In addition to plasma potency and the need for uniform screening approaches, early intervention is an equally significant factor to ensure CPT efficacy in treating COVID-19 patients. Joyner et al. [64] documented results from an Expanded Access Program for the treatment of COVID-19 patients with CPT. The study consisted of 35,322 patients spanning across 2807 acute care facilities in the US. While the program did not include a control group, results showed that transfusions within three days of COVID-19 diagnosis yielded an average reduction of 5% in mortality rates compared to those receiving transfusions after 4 or more days of diagnosis. Additionally, the trial by Zeng et al. [55] recorded the highest mortality rates across all studies (see Table 1) for both the CPT recipient (83%) and control (93%) groups. This study also reported the longest time for intervention compared to all existing trials—more than 21 days after diagnosis. Hence, CPT is generally not effective in reducing mortality in critically ill patients and is directly linked to the scalability of testing and contact tracing to ensure early intervention.

### 4.3. Donor–Recipient Risks

Among the reviewed articles, only Duan et al. [42] reported mild CPT side effects. However, previous studies on CPT therapy in patients infected by diseases other than COVID-19 have reported adverse (and occasionally severe) reactions. In general, the risk of a thrombotic event in CPT recipients can reach up to 15% [88]. More specifically, Luke et al. [19] conducted CPT trials on Spanish influenza (H5N1) patients who experienced tremors, facial spotting, fever, and transfusion-associated circulatory overload (TACO) [89]. Based on CPT trials on Ebola patients by Takada et al. [90], the transmitted antibodies caused complement binding to the virus, which strengthened the infection in recipient cells. Further research is needed to rule out the likelihood of this complement effect in COVID-19 patients. In terms of donor safety, CPT may cause mild and short-lived side effects, including dizziness, fatigue, and fainting associated with the loss of water and nutrients through the plasma extraction. Other CPT process-based inadequacies in terms of sterility or expertise may lead to bacterial or viral transmissions to a donor and, in less common cases, a fatal citrate reaction, which may trigger cardiac arrest [91].

### 4.4. Importance of Large-Scale RCTs

Despite the preliminary evidence on the efficacy of CPT for treating hospitalized COVID-19 patients, the lack of sufficient RCT data remains a critical issue. Many researchers refer to unsatisfactory results from RCTs on CPT in influenza A [92,93] and respiratory syncytial virus (RSV) [94] patients. While large-scale RCTs are understandably challenging given the relatively short duration since the emergence of COVID-19, they remain instrumental to reaching a scientific consensus regarding the efficacy of CPT. However, scalable RCTs warrant considerable investment and resources, which may be diverted away from other areas of the pandemic response [89]. For instance, testing large numbers of CPT donors as part of their screening process may reduce the rate of public health testing for contact tracing and early intervention. Hence, a careful optimization approach based on mathematical modeling is recommended to assess the magnitude of this resource diversion to scalable RCTs and the overall tradeoff.

An important limitation to the review is the merging of mortality rates across studies with different patient group sizes, in addition to biases from differences in the nation of study origin, concomitant treatment approaches, antibody titer, dosage volume, and duration of post-CPT patient follow-up. However, given the shortage in large-scale RCT data, the aggregation of data from multiple CPT trial formats provides important insights for both its preliminary efficacy and for the design of more representative and scalable RCTs.

## 5. Conclusions

Aggregated results show an overall reduction of 46% in mortality in patients receiving CPT therapy compared to control groups receiving standard supportive care and/or repurposed drugs. Despite the promising preliminary evidence from trials and case studies to date, several challenges remain in terms of the technical, safety, and resource aspects of CPT trials as presented in Section 4. Early intervention is shown to be a critical factor for CPT efficacy in treating COVID-19 patients, which is coupled to the scalability of testing and contact tracing. The recruitment of plasma donors, consistency of adequate ranges of SARS-CoV-2 antibody titer, risks of adverse effects to CPT donors/recipients, and robust management of healthcare resources are some of the major challenges to consider before implementing large-scale RCTs.

## Figures and Tables

**Table 1:** Studies showing effects of convalescent plasma therapy in COVID-19 cases.

Study, Country	CPT (and Control) Patient Count	CPT and (Control) Mortality (%)	CPT and (Control) Cohort Age Group	Antibody Threshold ^a^	Plasma Volume (mL)	Follow-Up Days
Randomized Clinical Trials (RCTs)						
[49], Spain	38; (43)	0; (9)	61; (60)	-	250–300	29
[51], Netherlands	43; (43)	14; (26)	63; (61)	NA(t) ≥ 1:80	≥300	30
[26], China	52; (51)	16; (24)	70; (69)	NA(t) ≥ 1:640	200	28
[50], Iraq	21; (28)	5; (29)	56; (48)	EIgG(i) > 1.25	≥200	30
Matched-Control Studies (MCSs)						
[61], Iran	115; (74)	15; (24)	54; (57)	EIgG(i) > 1.1	≥500	30
[63], Turkey	888; (888)	25; (28)	60; (61)	-	200–600	17
[56], USA	47; (1340)	23; (42)	59; (-)	NA(t) ≥ 1:500	≥200	30
[42], China	10; (10)	0; (30)	53; (53)	NA(t) ≥ 1:640	200	-
[54], USA	20; (20)	10; (30)	60; (-)	-	≥200	14
[64], USA	35,322; (-) ^b^	[8.3–26.7] ^c^; (-)	60; (-)	S/Co [4.62–18.45]	200	7
[59], USA	39; (156)	13; (24)	55; (54)	EIgG(t) ≥ 1:320	500	14
[53], Qatar	40; (40)	3; (13)	48; (56)	-	400	28
[52], Italy	46; (23)	7; (30)	63; (-)	NA(t) ≥ 1:80	≥250	7
[62], USA	64; (177)	13; (16)	61; (61)	-	≥200	28
[57], Argentina	868; (2298)	25; (44)	56; (64)	EIgG(i) > 1350	200–250	28
[58], USA	321; (582)	6; (12)	53; (60)	EIgG(i) ≥ 1350	≥200	60
[60], China	138; (1430)	2; (4)	65; (63)	-	≥200	14
[55], China	6; (15)	83; (93) ^d^	62; (73)	-	≥200	-
Case Reports (CRs)						
[70], South Korea	2	0	69	-	250	15
[75], USA	1	0	35	-	400	10
[76], China	1	0	38	-	300	31
[71], Hungary	2	0	-	-	600	14
[65], China	16	0	65	TA [10.9–115 AU/mL]	200–1200	8
[77], Turkey	1	0	55	EIgG(i) > 1.1	350	11
[80], USA	31	13	-	-	≥200	7
[79], France	17	6	58	NA(t) ≥ 1:40	800	7
[72], South Korea	1	0	68	-	500	23
[81], USA	29	17	58	-	200	28
[67], China	6	0	61	NA(t) ≥ 1:40	200	60
[68], USA	3	0	24	-	400	31
[78], China	1	0	100	NA(t) ≥ 1:640	300	13
[66], Mexico	8	0	57	EIgG(t) > 1:100	500	23
[82], Mexico	10	20	52	-	≥200	8
[73], China	1	0	66	EIgG(t) > 1:160	400	26
[43], China	5	0	65	NA(t) ≥ 1:40	400	47
[83], USA	24	42	69	EIgG(t) ≥ 1:320	500	9
[74], China	1	0	65	-	800	11
[84], China	6	0	58	-	≥200	25
[69], China	4	0	57	-	≥200	38

^a^ Different serologic tests to report antibody levels across studies, including the enzyme-linked immunosorbent assay for immunoglobulin G (EIgG) and neutralizing antibody (NA) indices (i) or titers (t), signal-to-cutoff ratio (S/Co), and total antibodies (TA) in arbitrary units per volume (AU/mL). ^b^ Joyner et al. [64] reports on an Expanded Access Program for the treatment of COVID-19 patients with CPT. The program did not include a control group and was, therefore, excluded from the boxplot and random-effects model. ^c^ Range of 7- and 30-day mortality rates corresponding to different times of transfusion (<3 or ≥4 days) after diagnosis and antibody S/Co levels. ^d^ The CPT and control group mortality rates (83 and 93%) reported by Zeng et al. [55] are not shown as outliers in Figure 1 for visualization purposes.

## Data Availability

All data incorporated in this review are presented and duly cited in Table 1.
